# Inanspruchnahme von Maßnahmen der betrieblichen Gesundheitsförderung in Deutschland – Ergebnisse der Studie „Gesundheit in Deutschland aktuell“ (GEDA 2014/2015-EHIS)

**DOI:** 10.1007/s00103-020-03239-z

**Published:** 2020-11-04

**Authors:** Sabine Ludwig, Anne Starker, Sophie Hermann, Susanne Jordan

**Affiliations:** 1 Abteilung für Epidemiologie und Gesundheitsmonitoring, Robert Koch-Institut, Berlin, Deutschland; 2 Department für Angewandte Gesundheitswissenschaften, Hochschule für Gesundheit Bochum, Gesundheitscampus 6–8, 44801 Bochum, Deutschland; 3 Institut für Medizinische Soziologie und Rehabilitationswissenschaften, Charité – Universitätsmedizin Berlin, Berlin, Deutschland

**Keywords:** Arbeit und Gesundheit, Kantine, Rückengesundheit, Stressbewältigung, Gesundheitsmonitoring, Occupational health, Canteen, Back health, Stress management, Health monitoring

## Abstract

**Hintergrund/Zielsetzung:**

Maßnahmen der betrieblichen Gesundheitsförderung (BGF) sollen die Arbeitsorganisation und die Arbeitsbedingungen verbessern sowie die persönlichen Kompetenzen der Beschäftigten fördern. Sie können einen großen Teil der Bevölkerung erreichen. Ziel der Studie ist, die Inanspruchnahme von verhältnis- und verhaltensbezogenen BGF-Maßnahmen anhand zentraler individueller und betrieblicher Faktoren zu beschreiben.

**Material und Methoden:**

In der repräsentativen bevölkerungsbasierten Querschnittsstudie „Gesundheit in Deutschland aktuell“ (GEDA 2014/2015-EHIS) des Robert Koch-Instituts wurden 14.389 Erwerbstätige im Alter von 18 bis 64 Jahren zur Kenntnis und Inanspruchnahme von Angeboten zu Rückengesundheit, Stressbewältigung/Entspannung und einer Kantine mit gesunden Ernährungsangeboten in den letzten 12 Monaten in ihrem Unternehmen befragt. Dabei wurde nach soziodemografischen Faktoren, Gesundheitsbewusstsein und dem subjektiven Gesundheitszustand stratifiziert.

**Ergebnisse:**

Das Angebot einer Kantine wird von 64,6 % der Frauen (F) und 66,2 % der Männer (M) genutzt. Angebote zur Rückengesundheit (F: 26,2 %; M: 18,7 %) und Stressbewältigung/Entspannung (F: 35,2 %; M: 25,6 %) werden deutlich weniger in Anspruch genommen. Beschäftigte mit ausgeprägtem Gesundheitsbewusstsein nutzen alle 3 Angebote häufiger als Beschäftigte mit weniger ausgeprägtem Gesundheitsbewusstsein. Männer mit schlechtem Gesundheitszustand nutzen die verhaltenspräventiven Angebote häufiger als Männer mit gutem Gesundheitszustand.

**Schlussfolgerung:**

Um weitere Erwerbstätige mit BGF-Maßnahmen zu erreichen, sollten diese zielgruppenspezifisch konzipiert werden. Geschlechter- und Altersaspekte, der Umfang der Erwerbstätigkeit, das Gesundheitsbewusstsein sowie der Gesundheitszustand sollten berücksichtigt werden.

## Abstract (en)

**Background/Objectives:**

Workplace health promotion (WHP) interventions can reach a large part of the population. They are designed to improve work organisation and conditions and to promote the personal competencies of employees. Here the aim was to describe the use of WHP interventions based on individual factors and factors related to the size and branch of the companies.

**Materials and methods:**

In the representative population-based study “German Health Update” (GEDA 2014/15-EHIS) conducted by the Robert Koch Institute, 14,389 employees aged between 18 and 64 years were asked about their knowledge and use of workplace measures in their companies during the last 12 months regarding back health, stress management/relaxation and a canteen with healthy food. In addition to socio-demographic factors, health awareness and self-rated health on the use of WHP interventions was analysed.

**Results:**

A canteen with healthy food is used by 64.6% of women (F) and 66.2% of men (M); offers for back health (F: 26.2%; M: 18.7%) and stress/relaxation (F: 35.2%; M: 25.6%) are used significantly less. Employees with more pronounced health awareness use the offers more frequently than employees with a less pronounced awareness of health. Men with poor self-rated health make more use of offers for back health and stress/relaxation than men with good self-rated health.

**Conclusions:**

In order to reach a larger part of the working population, WHP measures should take the needs of specific target groups into account including sex/gender and age aspects as well as the extent of employment, health awareness and self-rated health status.

## Einleitung

Der Einfluss der Arbeitswelt auf die Gesundheit ist vielfach belegt [1]. Der Arbeitsplatz kann neben einem Einkommen und psychosozialen Ressourcen auch Stress und gesundheitliche Belastungen für Beschäftigte bedeuten [2, 3]. Gleichzeitig bietet die Arbeitswelt gute Voraussetzungen für die Anwendung vorbeugender Maßnahmen zur Gesunderhaltung, da sich ein großer Teil der Bevölkerung regelmäßig in Betrieben oder Unternehmen aufhält. Zum Beispiel waren im Jahr 2018 in Deutschland rund 19,5 Mio. Frauen und 22,4 Mio. Männer erwerbstätig. Der Anteil der erwerbstätigen Frauen an der weiblichen Bevölkerung im erwerbsfähigen Alter lag bei 72,1 % und der entsprechende Anteil bei den Männern bei 79,6 % [4]. Zur Sicherung und Förderung von Gesundheit am Arbeitsplatz gibt es verschiedene Ansätze. Zum einen ist hier der gesetzlich vorgeschriebene Arbeitsschutz zu nennen, zum anderen führen Betriebe freiwillige vom Arbeitsgeber initiierte Maßnahmen wie das betriebliche Gesundheitsmanagement und die betriebliche Gesundheitsförderung (BGF) durch. Letztere steht im Fokus dieses Beitrags.

Unter BGF können nach der Luxemburger Deklaration zur betrieblichen Gesundheitsförderung in der Europäischen Union „alle gemeinsamen Maßnahmen von Arbeitgebern, Arbeitnehmern und der Gesellschaft zur Verbesserung von Gesundheit und Wohlbefinden am Arbeitsplatz“ gefasst werden [5]. Eine verbesserte Arbeitsorganisation, bessere Arbeitsbedingungen und Stärkung der persönlichen Kompetenzen der Beschäftigten sind das Ziel von BGF. Die Beteiligung der Beschäftigten ist bei der Entwicklung der Maßnahmen anzustreben [5]. Als eine freiwillige Leistung der Betriebe, die Arbeits- und Gesundheitsschutz ergänzt, kann BGF Bestandteil des betrieblichen Gesundheitsmanagements oder der betrieblichen Organisationsentwicklung sein, Mitarbeiter*innenbeteiligung umfassen oder sich auf Einzelmaßnahmen zur Verbesserung von konkreten Arbeitsbedingungen (Verhältnisse) oder des Gesundheitsverhaltens der Beschäftigten konzentrieren [6]. Neben den Betrieben sind die gesetzlichen Krankenkassen ein wichtiger Anbieter von BGF-Maßnahmen. Ihre Rolle wurde mit dem Inkrafttreten des Präventionsgesetzes im Jahr 2015 gestärkt, wobei die Kassenleistungen auch einen Beitrag zur Verminderung geschlechter- und sozial bedingter Ungleichheit von Gesundheitschancen leisten sollen [7]. Für die verschiedenen Handlungs- und Themenfelder der BGF liegt eine unterschiedlich starke Evidenz von zumeist kleinen bis moderaten Effekten auf ihre gesundheitsfördernde Wirkung vor, z. B. für die Steigerung körperlicher Aktivität, die Förderung gesunder Ernährung und die Rauchentwöhnung. Teilweise ist die Studienlage jedoch unzureichend, um die Evidenz beurteilen zu können, was besonders für verhältnispräventive Maßnahmen gilt [8]. Trotz der noch nicht befriedigenden Studienlage gilt die BGF insgesamt als vielversprechender Ansatz zur Erhaltung und Förderung der Gesundheit von Erwerbstätigen.

Daten über die Verbreitung von BGF in Deutschland liegen überwiegend aus Arbeitgeberbefragungen vor [9, 10], selten aus betrieblichen Mitarbeiterbefragungen oder bevölkerungsbezogenen Erhebungen der erwerbstätigen Bevölkerung [11]. Darüber hinaus gibt der jährlich erscheinende Präventionsbericht der gesetzlichen Krankenkassen einen Überblick über die von gesetzlichen Kassen erbrachten BGF-Leistungen anhand der von den Kassen ausgefüllten Dokumentationsbögen [12]. Die meisten Publikationen berücksichtigen Unternehmensgröße oder Wirtschaftsbranche, einige berichten über die Art der Angebote, wobei auch andere betriebliche Maßnahmen zur Förderung der Gesundheit, z. B. des Arbeitsschutzes, abgebildet werden. Soziodemografische Faktoren wie Geschlecht und Alter werden meist dargestellt, seltener Berufsstatus und Umfang der Erwerbstätigkeit. Forschungslücken bestehen hinsichtlich der Bedeutung von Gesundheitszustand und Gesundheitsbewusstsein der Beschäftigten bei der Teilnahme an Angeboten der BGF in Deutschland, und zwar besonders im Hinblick auf einzelne verhaltens- und verhältnisbezogene Maßnahmen. Dieser Beitrag soll diese Lücken schließen und beschreibt die Inanspruchnahme von BGF in Deutschland aus Sicht der Beschäftigten unter Berücksichtigung zentraler individueller und betrieblicher Faktoren [13]. Hierzu gehören Faktoren der Soziodemografie (Alter, Geschlecht), Beruf und Arbeitsplatz (Berufsstatus, Umfang der Erwerbstätigkeit), Gesundheitszustand, Einstellung zur Gesundheit (Gesundheitsbewusstsein) und Betrieb (Betriebsgröße und Branche). Untersucht werden dabei 3 verschiedene Angebote der BGF: das verhältnisorientierte Angebot einer Kantine mit gesunden Ernährungsangeboten und die verhaltensbezogenen Angebote zur Rückengesundheit und zur Stressbewältigung/Entspannung.

## Methoden

Datengrundlage für die vorliegenden Auswertungen ist die Studie „Gesundheit in Deutschland aktuell“ (GEDA 2014/2015-EHIS), die von November 2014 bis Juli 2015 stattfand. Auf Basis einer Einwohnermeldeamtsstichprobe wurden zufällig ausgewählte Personen ab einem Alter von 18 Jahren befragt, die ihren ständigen Wohnsitz in Deutschland hatten. Insgesamt beantworteten 24.016 Personen (13.144 Frauen, 10.872 Männer) den schriftlich oder online auszufüllenden Fragebogen. Konzept und Methoden von GEDA 2014/2015-EHIS wurden an anderer Stelle bereits ausführlich beschrieben [14, 15].

Die Analysestichprobe umfasst alle Personen im Alter zwischen 18 und 64 Jahren, die auf die Frage: „Welche Lebenssituation trifft derzeit überwiegend auf Sie zu?“, antworteten, dass sie in Vollzeit, Teilzeit, Altersteilzeit oder geringfügig erwerbstätig sind, und die zusätzlich auf die Frage: „Welche berufliche Stellung haben Sie in Ihrer Haupterwerbstätigkeit?“, antworteten, dass sie Angestellte*r, Arbeiter*in oder Beamtin*Beamter (auch Anwärter*in) sind (*n* = 12.072). Von der Untersuchung ausgeschlossen wurden alle nichterwerbstätigen Personen sowie Personen die angaben, ein freiwilliges soziales/ökologisches/kulturelles Jahr zu leisten oder freiwillig Wehrdienst- oder Bundesfreiwilligendienstleistende*r zu sein. Außerdem nicht berücksichtigt wurden Studienteilnehmer*innen, die angaben, Landwirt*in im Haupterwerb, selbstständig (mit und ohne Mitarbeiter*innen), mithelfende Familienangehörige oder Auszubildende*r (auch Praktikant*in, Volontär*in) zu sein.

Die Inanspruchnahme der BGF wird zu folgenden Angeboten untersucht: Kantine mit gesunden Ernährungsangeboten, Rückengesundheit und Stressbewältigung/Entspannung. Die Fragen zur BGF sind an Items anderer Studien in Deutschland angelehnt, die mittels repräsentativer telefonischer Befragungen in der erwerbstätigen Bevölkerung vom Wissenschaftlichen Institut der AOK (WIdO) und der Initiative Gesundheit und Arbeit (iga) durchgeführt wurden, zumeist im Zusammenhang mit Themen wie Arbeitsbelastungen und Arbeitsschutz [11, 16, 17].

In GEDA 2014/2015-EHIS wurde zunächst die Kenntnis über die jeweiligen Angebote mit folgender Eingangsfrage erfasst (Antwortkategorien: Ja/Nein/weiß nicht): „Gab es in Ihrem Betrieb/Ihrem Unternehmen in den letzten 12 Monaten …

- … eine Kantine mit gesunden Ernährungsangeboten (z. B. tägliches Angebot von Gemüse und frischem Salat, täglich fleischlose Gerichte, regelmäßiges Angebot von Pell- oder Folienkartoffeln)?
- … ein Angebot zur Rückengesundheit (z. B. Rückenschule, Rückengymnastik)?
- … ein Angebot zur Stressbewältigung/Entspannung (z. B. Zeitmanagement, autogenes Training)?“

Bei Bejahung der Eingangsfrage wurde die Inanspruchnahme erfragt: „Haben Sie dieses Angebot in Anspruch genommen?“ (Antwortkategorien: Ja/Nein; [18]).

Um Unterschiede der Inanspruchnahme von BGF-Angeboten differenziert abzubilden, wurden verschiedene soziodemografische Faktoren, Aspekte der beruflichen Tätigkeit, das Gesundheitsbewusstsein und der subjektiv eingeschätzte Gesundheitszustand erhoben.

Der Berufsstatus als wichtiger sozioökonomischer Indikator der beruflichen Lage steht in engem Zusammenhang mit Gesundheit [19, 20]. Zur Bestimmung des individuellen Berufsstatus werden das Einkommen und die Qualifikation der Beschäftigten in den nach der „Klassifikation der Berufe 2010“ codierten beruflichen Tätigkeiten herangezogen [21]. Als Kriterium für die Zuweisung von Punktwerten diente dabei der International Socio-Economic-Index of Occupational Status (ISEI) nach [22]. Für die Analysen wurden die Punktwerte der gewichteten Fälle von GEDA 2014/2015-EHIS in Quintile eingeteilt. Verglichen werden die unteren 20 % (niedriger individueller Berufsstatus) mit den mittleren 60 % (mittlerer individueller Berufsstatus) und den oberen 20 % (hoher individueller Berufsstatus).

Bei der Inanspruchnahme von BGF-Maßnahmen spielt auch die Betriebsbranche eine wichtige Rolle. [23, 24]. Der Wirtschaftsbereich bzw. die Branche wurde mit der Aufforderung erfasst: „Bitte ordnen Sie den Betrieb, in dem Sie tätig sind, einer Branche/einem Wirtschaftszweig zu.“ Die vorgegebenen Antwortkategorien orientieren sich an der Abfrage der Branchen/Wirtschaftszweige in der EU-Statistik über Einkommen und Lebensbedingungen 2013 (EU Statistics on Income and Living Conditions, EU-SILC; [25]). Für die Analysen wurden die häufigsten 5 Branchen/Wirtschaftszweige dargestellt, denen 58,6 % der befragten Erwerbstätigen unter 65 Jahren zugeordnet werden können. Dies sind: 1) verarbeitendes Gewerbe/Herstellung von Waren; 2) Groß- und Einzelhandel, Instandhaltung und Reparatur von Kraftfahrzeugen; 3) Verwaltung, Gerichte, öffentliche Sicherheit und Ordnung, Verteidigung, Sozialversicherung; 4) Erziehung und Unterricht sowie 5) Gesundheits- und Sozialwesen.

Zur Erfassung der Betriebsgröße wurde im Anschluss an die Frage nach der Branche/Wirtschaftszweig folgende Frage gestellt: „Wie viele Personen arbeiten in diesem Betrieb?“ (Antwortkategorien: bis einschließlich 9 Personen/10 bis einschließlich 49 Personen/50 Personen oder mehr).

Aus der Forschung ist bekannt, dass die Teilnahme an präventiven Maßnahmen mit dem Gesundheitsbewusstsein assoziiert sein kann [26]. Das Gesundheitsbewusstsein wurde mit der Frage ermittelt: „Wie stark achten Sie im Allgemeinen auf Ihre Gesundheit?“ (sehr stark/stark/mittelmäßig/weniger stark/gar nicht; [27]). Für die Auswertungen werden die zusammengefassten Antworten „sehr stark/stark“ und „mittelmäßig/weniger stark/gar nicht“ ausgewiesen. Der subjektive Gesundheitszustand erfasst das persönliche Wohlbefinden und spielt eine Rolle hinsichtlich der künftigen Inanspruchnahme medizinischer Leistungen [28]. Die subjektive Gesundheit wurde mittels folgender Frage erfasst: „Wie ist Ihr Gesundheitszustand im Allgemeinen?“ (Antwortkategorien: sehr gut/gut/mittelmäßig/schlecht/sehr schlecht; zusammengefasste Antworten: sehr gut/gut sowie mittelmäßig/schlecht/sehr schlecht).

Alle Analysen wurden mit dem Statistikpaket Stata SE 15.1 für erwerbstätige Studienteilnehmer*innen im Alter zwischen 18 und 64 Jahren durchgeführt und wurden mit einem Gewichtungsfaktor berechnet. Dieser korrigiert die Abweichungen der Stichprobe von der Bevölkerungsstruktur (Stand: 31.12.2014) hinsichtlich Geschlecht, Alter, Kreistyp und Bildung, wobei der Kreistyp den Grad der Urbanisierung widerspiegelt und der regionalen Verteilung in Deutschland entspricht. Gruppenunterschiede wurden mit dem Chi-Quadrat-Test für komplexe Stichproben auf Signifikanz geprüft. Von einem signifikanten Unterschied wird ausgegangen, wenn der unter Berücksichtigung der Gewichtung und des Survey-Designs berechnete *p*-Wert kleiner als 0,05 ist.

## Ergebnisse

### Beschreibung der Untersuchungsstichprobe

Die Tab. 1 gibt einen Überblick über zentrale Merkmale der Studienpopulation. Dabei sind sowohl die ungewichteten absoluten Häufigkeiten als auch die relativen Häufigkeiten dargestellt. Für die gewichteten Prozentwerte sind die jeweiligen 95 %-Konfidenzintervalle (KI) angegeben.

**Table 1:** 

		*n* ungew.	% gewichtet	95 %-KI
Geschlecht	Weiblich	6610	47,5	46,4–48,6
	Männlich	5462	52,5	51,4–53,6
Alter	18–29 Jahre	1662	15,8	15,0–16,7
	30–44 Jahre	4122	34,1	33,0–35,1
	45–64 Jahre	6288	50,1	49,0–51,2
Berufsstatus (ISEI)	Niedrig	1957	21,0	20,0–22,0
	Mittel	6761	60,5	59,4–61,6
	Hoch	2722	18,5	17,8–19,3
	Fehlende Werte (Missings)	632	–	–
Betriebsgröße	Kleinstbetrieb (1–9 Personen)	1778	15,3	14,5–16,1
	Kleinunternehmen (10–49 Personen)	3223	27,1	26,1–28,1
	Mittleres bis großes Unternehmen (>50 Personen)	6817	57,6	56,5–58,7
	Fehlende Werte (Missings)	194	–	–
Branche	Verarbeitendes Gewerbe/Herstellung von Waren	1767	17,8	16,9–18,7
	Groß- und Einzelhandel, Instandhaltung, Reparatur von Kraftfahrzeugen	1032	10,1	9,4–10,8
	Verwaltung, Gerichte, öffentl. Sicherheit/Ordnung, Verteidigung, Sozialversicherung	1185	9,5	8,9–10,2
	Erziehung und Unterricht	1164	7,0	6,5–7,5
	Gesundheits- und Sozialwesen	1967	14,6	13,8–15,3
	Andere Branchen	4432	41,0	39,9–42,1
	Fehlende Werte (Missings)	525	–	–
Umfang der Erwerbstätigkeit	Teilzeit	3681	28,2	27,3–29,2
	Vollzeit	8391	71,8	70,8–72,7
	Fehlende Werte (Missings)	0	–	–
Subjektiver Gesundheitszustand	Sehr gut	2202	17,4	16,6–18,2
	Gut	7281	60,2	59,1–61,3
	Mittelmäßig	2323	20,3	19,4–21,2
	Schlecht	210	1,9	1,6–2,2
	Sehr schlecht	18	0,2	0,1–0,3
	Fehlende Werte (Missings)	38	–	–
Subjektiver Gesundheitszustand (aggr.)	Sehr gut bis gut	9483	77,6	76,7–78,5
	Mittelmäßig bis sehr schlecht	2551	22,4	21,5–23,3
Gesundheitsbewusstsein	Sehr stark	813	6,6	6,1–7,2
	Stark	4590	36,1	35,1–37,1
	Mittelmäßig	5757	49,3	48,2–50,4
	Weniger stark	770	6,9	6,4–7,6
	Gar nicht	100	1,1	0,8–1,3
	Fehlende Werte (Missings)	42	–	–
Gesundheitsbewusstsein (aggregiert)	Stark bis sehr stark	5403	42,7	41,6–43,8
	Weniger stark bis gar nicht	6627	57,3	56,2–58,4

*KI* Konfidenzintervalle, *ungew.* ungewichtet

### Inanspruchnahme einer Kantine mit gesunden Ernährungsangeboten

Von den Frauen und Männern, die Kenntnis von einer Kantine hatten (Frauen: 27,7 %; Männer: 36,5 %), nahm ein Anteil von 64,3 % der Frauen und 65,5 % der Männer eine Kantine mit gesunden Ernährungsangeboten in ihrem Betrieb in Anspruch (Tab. 2). Von den hier dargestellten Angeboten der betrieblichen Gesundheitsförderung wurde dieses Angebot von beiden Geschlechtern am häufigsten genutzt. Zwischen den Geschlechtern bestand kein signifikanter Unterschied.

**Table 2:** 

Kantine mit gesunden Ernährungsangeboten		Frauen			Männer		
		%	95 %-KI	*p*-Wert	%	95 %-KI	*p*-Wert
*Inanspruchnahme gesamt*		*64,3*	*61,5–67,0*	*–*	*65,5*	*62,7–68,2*	*–*
Alter	18–29 Jahre	72,4	65,5–78,4	*0,003*	70,4	63,0–76,9	*0,005*
	30–44 Jahre	67,3	61,8–72,3		69,7	65,4–73,7	
	45–64 Jahre	59,8	56,0–63,4		60,9	56,8–64,8	
Berufsstatus (ISEI)	Niedrig	67,3	57,9–75,5	*0,040*	62,0	53,4–69,8	*0,000*
	Mittel	61,5	58,1–64,9		60,8	57,0–64,4	
	Hoch	69,6	64,3–74,4		75,8	71,5–79,7	
Betriebsgröße	Kleinstbetrieb (1–9 Personen)	73,5	60,4–83,5	*0,041*	78,5	71,4–92,6	0,481
	Kleinunternehmen (10–49 Personen)	70,0	63,4–75,8		67,2	56,9–76,0	
	Mittleres bis großes Unternehmen (>50 Personen)	62,5	59,3–65,6		65,2	62,3–68,0	
Branche	Verarbeitendes Gewerbe/ Herstellung von Waren	64,5	56,7–71,6	0,416	63,6	58,3–68,5	0,578
	Groß- und Einzelhandel, Instandhaltung und Reparatur von Kraftfahrzeugen	67,1	54,1–78,0		70,4	57,5–80,6	
	Verwaltung, Gerichte, öffentliche Sicherheit/ Ordnung, Verteidigung, Sozialversicherung	60,3	51,5–68,5		63,5	55,0–71,2	
	Erziehung und Unterricht	62,5	55,7–68,9		71,6	61,3–80,1	
	Gesundheits- und Sozialwesen	57,4	51,6–63,0		64,7	56,7–72,0	
Umfang Erwerbstätigkeit	Teilzeit	57,9	53,4–62,3	*0,000*	61,1	49,1–71,9	0,410
	Vollzeit	69,0	65,6–72,2		65,8	63,0–68,4	
Subjektiver Gesundheitszustand	Sehr gut bis gut	63,6	60,4–66,8	0,377	67,3	64,4–70,2	*0,009*
	Mittelmäßig bis sehr schlecht	66,6	60,7–72,1		57,3	50,0–64,3	
Gesundheitsbewusstsein	Stark bis sehr stark	62,5	58,3–66,5	0,230	66,8	62,9–70,5	0,407
	Weniger stark bis gar nicht	65,7	62,1–69,1		64,6	60,8–68,3	

Bezogen auf alle Personen, die eine Kantine mit gesunden Ernährungsangeboten in ihrem Betrieb kennen

*KI* Konfidenzintervalle, *ungew.* ungewichtet, *p* Signifikanzniveau

Im Vergleich der Altersgruppen zeigt sich, dass sowohl Frauen (*p* = 0,003) als auch Männer (*p* = 0,005) in der Altersgruppe der 45- bis 64-Jährigen seltener eine Kantine mit gesunden Ernährungsangeboten in Anspruch nahmen als Frauen und Männer der jüngeren Altersgruppen. Während Männer mit hohem beruflichen Status das Angebot häufiger nutzten als Männer mit mittlerem und niedrigem beruflichen Status (*p* = 0,000), nutzten Frauen mit einem niedrigen oder hohen beruflichen Status das Angebot häufiger als Frauen mit mittlerem beruflichen Status (*p* = 0,040).

Eine Kantine mit gesunden Ernährungsangeboten wurde in Kleinstbetrieben von weiblichen Beschäftigten häufiger in Anspruch genommen als in kleinen, mittleren oder großen Betrieben (*p* = 0,041). Bei den männlichen Beschäftigten zeigten sich keine Unterschiede in der Inanspruchnahme nach Betriebsgröße. Auch im Vergleich der Branchen konnten sowohl bei den Frauen als auch bei den Männern keine Unterschiede zwischen den Gruppen festgestellt werden.

Im Umfang der Erwerbstätigkeit zeigten sich nur bei den Frauen Unterschiede: Hier gaben Frauen, die in Teilzeit beschäftigt waren, seltener an, eine Kantine mit gesunden Ernährungsangeboten zu nutzen, als Frauen mit einer Vollzeitbeschäftigung (*p* = 0,000).

Hinsichtlich des Gesundheitsbewusstseins zeigten sich für beide Geschlechter keine Unterschiede zwischen erwerbstätigen Personen, die angaben, gar nicht bis weniger stark auf ihre Gesundheit zu achten, und Personen, die angaben, stark bis sehr stark auf ihre Gesundheit zu achten. Hingegen nutzten Männer, die ihren Gesundheitszustand selbst als sehr gut bis gut einschätzten, häufiger eine Kantine mit gesunden Ernährungsangeboten als Männer, die diesen als mittel bis sehr schlecht einschätzten (*p* = 0,009). Bei den Frauen lagen keine signifikanten Gruppenunterschiede vor.

### Inanspruchnahme von Angeboten zur Rückengesundheit und zur Stressbewältigung/Entspannung

Kenntnis von einem Angebot zur Rückengesundheit hatten 32,0 % der Männer und 24,3 % der Frauen. Davon nahmen 25,5 % der Frauen und 18,1 % der Männer das Angebot in Anspruch (Tab. 3). Von den Frauen und Männern, die von Angeboten zur Stressbewältigung/Entspannung wussten (Frauen: 21,4 %; Männer: 24,3 %), nutzten 34,1 % der Frauen und 24,5 % der Männer das Angebot (Tab. 4).

**Table 3:** 

Angebot zur Rückengesundheit		Frauen			Männer		
		%	95 %-KI	*p*-Wert	%	95 %-KI	*p*-Wert
*Inanspruchnahme gesamt*		*25,5*	*22,8–28,4*	*–*	*18,1*	*16,0–20,5*	*–*
Alter	18–29 Jahre	24,4	18,4–31,6	0,533	13,6	8,5–21,0	0,329
	30–44 Jahre	23,8	19,6–28,6		18,7	14,8–23,4	
	45–64 Jahre	27,0	23,0–31,4		18,9	16,3–21,9	
Berufsstatus (ISEI)	Niedrig	26,0	16,5–38,5	0,763	22,1	14,5–32,0	0,263
	Mittel	26,0	23,0–29,3		18,2	15,4–21,3	
	Hoch	23,6	18,8–29,2		15,2	11,8–19,2	
Betriebsgröße	Kleinstbetrieb (1–9 Personen)	33,0	20,0–49,2	*0,006*	31,3	15,4–53,2	*0,042*
	Kleinunternehmen (10–49 Personen)	34,8	27,9–42,4		23,6	17,6–30,8	
	Mittleres bis großes Unternehmen (>50 Personen)	23,2	20,5–26,2		17,3	15,0–19,8	
Branche	Verarbeitendes Gewerbe/Herstellung von Waren	27,1	19,7–36,1	0,227	16,1	12,5–20,4	0,827
	Groß- und Einzelhandel, Instandhaltung und Reparatur von Kraftfahrzeugen	29,8	18,0–45,1		20,1	10,9–34,2	
	Verwaltung, Gerichte, öffentliche Sicherheit/ Ordnung, Verteidigung, Sozialversicherung	26,4	20,4–33,3		16,3	12,1–21,7	
	Erziehung und Unterricht	34,0	23,5–46,4		22,1	12,5–36,2	
	Gesundheits- und Sozialwesen	20,9	16,2–26,6		17,9	11,5–26,9	
Umfang Erwerbstätigkeit	Teilzeit	22,3	18,3–26,8	*0,040*	17,5	10,4–27,9	0,886
	Vollzeit	28,0	24,6–31,6		18,2	15,9–20,7	
Subjektiver Gesundheitszustand	Sehr gut bis gut	25,8	22,8–29,1	0,696	17,0	14,7–19,6	*0,047*
	Mittelmäßig bis sehr schlecht	24,4	18,8–31,1		23,0	17,7–29,4	
Gesundheitsbewusstsein	Stark bis sehr stark	29,6	25,7–33,8	*0,002*	19,9	16,6–23,7	0,183
	Weniger stark bis gar nicht	21,3	18,0–25,1		17,6	13,9–20,0	

Bezogen auf alle Personen, die mindestens ein Angebot zu Rückengesundheit in ihrem Betrieb kennen

*KI* Konfidenzintervalle, *ungew.* ungewichtet, *p* Signifikanzniveau

**Table 4:** 

Angebot zur Stressbewältigung/Entspannung		Frauen			Männer		
		%	95 %-KI	*p*-Wert	%	95 %-KI	*p*-Wert
*Inanspruchnahme gesamt*		*34,1*	*31,1–37,4*	*–*	*24,5*	*21,7–27,7*	**–**
Alter	18–29 Jahre	34,9	26,4–44,5	0,266	24,4	16,8–34,0	0,539
	30–44 Jahre	30,9	26,2–36,0		22,6	18,4–27,5	
	45–64 Jahre	36,5	32,3–40,9		26,0	22,3–30,1	
Berufsstatus (ISEI)	Niedrig	39,3	21,5–60,4	0,729	32,8	21,0–47,1	0,180
	Mittel	33,0	29,4–36,9		22,9	19,3–27,0	
	Hoch	34,7	29,1–40,7		26,4	22,0–31,4	
Betriebsgröße	Kleinstbetrieb (1–9 Personen)	40,0	26,9–54,7	*0,017*	40,4	20,7–63,8	*0,002*
	Kleinunternehmen (10–49 Personen)	42,3	35,0–49,9		37,6	28,8–47,4	
	Mittleres bis großes Unternehmen (>50 Personen)	31,4	28,1–35,0		22,5	19,6–25,8	
Branche	Verarbeitendes Gewerbe/ Herstellung von Waren	39,6	29,6–50,6	*0,020*	17,8	13,1–23,8	*0,031*
	Groß- und Einzelhandel, Instandhaltung und Reparatur von Kraftfahrzeugen	19,2	8,6–37,5		38,9	23,0–57,5	
	Verwaltung, Gerichte, öffentliche Sicherheit/ Ordnung, Verteidigung, Sozialversicherung	29,2	23,1–36,0		26,0	20,4–32,5	
	Erziehung und Unterricht	44,2	35,7–53,2		25,9	17,3–37,0	
	Gesundheits- und Sozialwesen	32,4	26,6–38,7		20,5	13,1–30,5	
Umfang Erwerbstätigkeit	Teilzeit	33,6	28,9–38,7	0,799	36,3	24,7–49,6	*0,040*
	Vollzeit	34,5	30,5–38,7		23,8	20,8–27,1	
Subjektiver Gesundheitszustand	Sehr gut bis gut	33,2	29,8–36,9	0,195	23,4	20,3–26,8	0,091
	Mittelmäßig bis sehr schlecht	38,3	31,6–45,5		30,6	23,1–39,3	
Gesundheitsbewusstsein	Stark bis sehr stark	36,0	32,0–40,2	0,164	29,6	25,2–34,4	*0,002*
	Weniger stark bis gar nicht	32,0	27,6–36,6		20,4	17,0–24,3	

Bezogen auf alle Personen, die mindestens ein Angebot zur Stressbewältigung/Entspannung in ihrem Betrieb kennen

*KI* Konfidenzintervalle, *ungew.* ungewichtet, *p* Signifikanzniveau

Insgesamt wurden damit sowohl die Angebote zur Rückengesundheit (*p* = 0,001) als auch die Angebote zur Stressbewältigung/Entspannung (*p* = 0,001) häufiger von Frauen als von Männern in Anspruch genommen. Für beide Angebote zeigten sich keine signifikanten Unterschiede zwischen den Altersgruppen. Dies traf für beide Geschlechter zu.

Für das Angebot zur Stressbewältigung/Entspannung zeigte sich nur für Männer ein signifikanter Unterschied hinsichtlich des Umfangs der Erwerbstätigkeit: Im Unterschied zu Männern, die in Vollzeit erwerbstätig waren, zeigte sich eine höhere Inanspruchnahme in der Gruppe der männlichen Erwerbstätigen in Teilzeit (*p* = 0,040). Für das Angebot zur Rückengesundheit hingegen zeigte sich, dass Frauen, die in Teilzeit beschäftigt waren, seltener ein solches Angebot nutzten als in Vollzeit erwerbstätige Frauen (*p* = 0,040).

Die betrieblichen Maßnahmen zur Rückengesundheit bzw. zur Stressbewältigung/Entspannung nutzten Frauen (*p* = 0,006 bzw. *p* = 0,017) und Männer (*p* = 0,042 bzw. *p* = 0,002) in Kleinstbetrieben bzw. kleinen Betrieben häufiger als in mittleren und großen Betrieben. Während für die Inanspruchnahme von Angeboten zur Rückengesundheit bei den Branchen kein signifikanter Unterschied vorlag, zeigte sich dieser hingegen für Angebote zur Stressbewältigung/Entspannung: Frauen aus dem Bereich Erziehung und Unterricht sowie Frauen aus dem verarbeitenden Gewerbe und Männer, die in den Bereichen Groß- und Einzelhandel, Instandhaltung und Reparatur von Kraftfahrzeugen tätig waren, nutzten die Angebote häufiger als Frauen (*p* = 0,020) bzw. Männer (*p* = 0,031) anderer Branchen.

Bei Betrachtung des subjektiven Gesundheitszustandes sowie des Gesundheitsbewusstseins zeigte sich einerseits, dass Männer mit einem mittleren bis schlechten subjektiven Gesundheitszustand häufiger ein Angebot zur Rückengesundheit nutzten als Männer, die ihren Gesundheitszustand als sehr gut bis gut einschätzten (*p* = 0,047). Zudem nahmen Männer, die angaben, stark bis sehr stark auf ihre Gesundheit zu achten, häufiger an Angeboten zur Stressbewältigung/Entspannung teil als Männer mit geringer ausgeprägtem Gesundheitsbewusstsein (*p* = 0,002). Bei den weiblichen Erwerbstätigen zeigten sich signifikante Gruppenunterschiede beim Gesundheitsbewusstsein: Frauen mit einem stark bis sehr stark ausgeprägten Gesundheitsbewusstsein nutzten die Angebote zur Rückengesundheit häufiger als Frauen mit einem weniger ausgeprägten Gesundheitsbewusstsein (*p* = 0,002).

## Diskussion

Insgesamt nutzen rund zwei Drittel der Befragten das verhältnispräventive Angebot einer Kantine mit gesunden Ernährungsangeboten; die verhaltensbezogenen Angebote zur Rückengesundheit und Stressbewältigung/Entspannung werden hingegen von nur rund 20–30 % der Befragten in Anspruch genommen. Die Daten des Fehlzeiten-Reports 2008 zeigen ebenfalls, dass rund zwei Drittel der Beschäftigten eine Kantine mit gesunden Ernährungsangeboten in Anspruch nehmen [11]. Die Ergebnisse des iga-Reports 12 von 2007 zeigen hingegen eine etwas höhere Inanspruchnahme von Angeboten zur Rückengesundheit (39 %) und zur Stressbewältigung (41 %) und eine etwas geringere Inanspruchnahme von Angeboten zum Thema gesunde Ernährung (56 %); zur Nutzung einer Kantine werden dort keine Aussagen gemacht [16]. Die Erwerbstätigenbefragung des Bundesinstituts für Berufsbildung (BIBB) und der Bundesanstalt für Arbeitsschutz und Arbeitsmedizin (BAuA) von 2011/2012 (BIBB/BAuA-Erwerbstätigenbefragung) zeigt insgesamt einen Anstieg der Inanspruchnahme von Maßnahmen, untersucht aber keine themenspezifischen Angebote der BGF [29].

Hinsichtlich der einzelnen Faktoren der Inanspruchnahme fällt auf, dass, betrachtet man mögliche Geschlechterunterschiede, Frauen die beiden verhaltenspräventiven Angebote zur Rückengesundheit und zur Stressbewältigung/Entspannung häufiger nutzen als Männer. Auch der Fehlzeiten-Report 2008 zeigt eine höhere Inanspruchnahme beider Angebote durch Frauen [11]. Die Ergebnisse des iga–Reports 12 zeigen hingegen, dass zwar mehr Frauen Angebote zur Rückengesundheit wahrnehmen, jedoch bei Angeboten zur Stressbewältigung/Entspannung kaum Geschlechterunterschiede vorliegen [16]. Die BIBB/BAuA-Erwerbstätigenbefragung findet für alle Maßnahmen keine bedeutenden Geschlechterunterschiede, allerdings wird dabei nicht nach den einzelnen Angeboten differenziert [29]. Als mögliche Begründung für die höhere Inanspruchnahme wird diskutiert, dass Frauen in der Regel mehr auf ihre Gesundheit achten als Männer. Dies zeigt sich u. a. an der allgemeinen Gesundheitsorientierung des Gesundheitsverhaltens und der damit verbundenen allgemein beobachtbaren höheren Inanspruchnahme von präventiven Angeboten von Frauen [26, 30–32].

Bei der Inanspruchnahme einer Kantine mit gesunden Ernährungsangeboten zeigen sich keine Geschlechterunterschiede, was zu erwarten gewesen wäre, da sich Frauen häufiger gesünder ernähren als Männer und ein besseres Wissen hinsichtlich gesunder Ernährung haben [33–37]. Unterschiede zeigen sich aber hinsichtlich des Umfangs der Erwerbstätigkeit: Mehr Frauen, die Vollzeit arbeiten, nehmen die Kantine in Anspruch als Frauen, die Teilzeit erwerbstätig sind. Dies trifft auch für die beiden anderen Angebote zu. Da ein größerer Teil der Frauen Teilzeit arbeitet, um Familie und Beruf besser vereinbaren zu können, insbesondere in der Altersgruppe zwischen 30 und 44 Jahren, könnte es sein, dass diese Zielgruppe schlechter von Maßnahmen der betrieblichen Gesundheitsförderung erreicht wird (Zeitmangel, Angebote außerhalb der Arbeitszeit) [38–41]. Die Daten des Fehlzeiten-Reports 2008 zeigen, dass etwas mehr Männer das Angebot einer Kantine nutzen (M: 70,5 %; F: 62,2 %); [11].

Hinsichtlich möglicher Altersunterschiede zeigen unsere Ergebnisse, dass weniger Beschäftigte in der Gruppe der 45- bis 64-Jährigen eine Kantine in Anspruch nahmen. Das bestätigen auch die Daten des Fehlzeiten-Reports 2008 [11] und die BIBB/BAuA-Erwerbstätigenbefragung von 2011/2012 [29]. Das könnte darauf hinweisen, dass die Angebote weniger auf die Bedürfnisse dieser Altersgruppe zugeschnitten sind. Etwas mehr Beschäftigte nehmen die Angebote zur Rückengesundheit und zur Stressbewältigung/Entspannung wahr, die Unterschiede sind aber nicht signifikant. Die Daten des iga-Reports 12 zeigen hingegen, dass deutlich mehr Beschäftigte über 60 Jahren die Angebote zur Stressbewältigung in Anspruch nehmen [16]. Dies könnte daran liegen, dass mit fortgeschrittenem Alter die chronischen Erkrankungen zunehmen sowie die durch die Arbeit empfundene Stressbelastung [42].

Für den beruflichen Status zeigen auch andere Studien einen Zusammenhang zwischen der Inanspruchnahme von Angeboten der Prävention und Gesundheitsförderung, wonach Frauen und Männer mit hohem sozioökonomischen Status häufiger an Angeboten zur Gesundheitsförderung und Prävention teilnehmen und sich in der Regel auch gesünder ernähren [13, 26, 33]. Die Ergebnisse der BIBB/BAuA-Erwerbstätigenbefragung von 2011/2012 zeigen insgesamt keine signifikanten Unterschiede in der Inanspruchnahme zwischen Arbeiter*innen, Angestellten und Beamten [29].

Die Betriebsgröße scheint eine Rolle für die Inanspruchnahme von BGF-Maßnahmen zu spielen: An den drei dargestellten Angeboten nehmen mehr Beschäftigte in Kleinstbetrieben die Angebote in Anspruch als in kleinen, mittleren oder großen Betrieben. Dabei ist jedoch zu beachten, dass die Anzahl der Kleinstbetriebe gering war und die Repräsentativität der Daten unter Umständen einschränkt ist. Das Angebot an Maßnahmen ist jedoch insgesamt geringer. Das bestätigen auch die anderen Studien [16, 29]. Gründe hierfür könnten sein, dass in Kleinstunternehmen die Produktions- und Dienstleistungsprozesse weniger arbeitsteilig sind und daher kürzere Informationswege bestehen. Die Hierarchien sind flacher und es besteht eine größere soziale Nähe zwischen Führungskräften und Beschäftigten. Dies könnte dazu führen, dass die Beschäftigten in der Regel eher Kenntnis über die Angebote zur betrieblichen Gesundheitsförderung haben. Zudem wird davon ausgegangen, dass in kleinen Betrieben die Angebote für alle zugänglich sind, wobei in größeren Betrieben Maßnahmen oftmals nur für bestimmte Arbeitsbereiche konzipiert sind [16, 29].

Hinsichtlich der Branche ist nicht bei allen drei Angeboten ein enger Zusammenhang mit der Inanspruchnahme zu erkennen: So bestand bei der Branche kein Zusammenhang mit der Inanspruchnahme einer Kantine oder auf Angebote zur Rückengesundheit. Dies traf für beide Geschlechter zu. Angebote zur Stressbewältigung/Entspannung werden jedoch von Frauen aus dem verarbeitenden Gewerbe und aus dem Bereich Erziehung und Unterricht häufiger in Anspruch genommen sowie von Männern, die in den Bereichen Groß- und Einzelhandel, Instandhaltung und Reparatur von Kraftfahrzeugen tätig sind. Die anderen Studien finden andere Ergebnisse, entscheidend scheint aber die Arbeitsbelastung zu sein [11].

Beschäftigte mit stärker ausgeprägtem Gesundheitsbewusstsein nehmen die Maßnahmen häufiger in Anspruch. Dies trifft für beide Geschlechter zu. Signifikant sind die Unterschiede für Angebote zur Stressbewältigung bei den Männern und für Angebote zur Rückengesundheit bei den Frauen. Dies wird auch durch andere Studien und Theorien des Gesundheitsverhaltens gestützt, die sich allerdings nicht spezifisch auf BGF-Maßnahmen beziehen: So beeinflusst die Erwartung, dass das individuelle Handeln positive Auswirkungen auf die Gesundheit haben kann, das Gesundheitsverhalten und somit die Nutzung von präventiven Maßnahmen [26, 43, 44].

Bei Männern spielte der subjektive Gesundheitszustand eine wichtige Rolle hinsichtlich der Inanspruchnahme von zwei Angeboten: So nutzten Männer mit gutem bis sehr guten Gesundheitszustand häufiger das Angebot einer Kantine mit gesunden Ernährungsangeboten als Männer, die diesen als mittel bis sehr schlecht einschätzten. Angebote zur Rückengesundheit und zur Stressbewältigung/Entspannung hingegen wurden häufiger von Männern mit einem mittleren bis schlechten subjektiven Gesundheitszustand genutzt. Dieses Ergebnis entspricht zentralen Annahmen bekannter Modelle des Gesundheitsverhaltens [44, 45]: So begünstigen das selbst eingeschätzte Risiko für eine Erkrankung bzw. dessen Schweregrad präventives Gesundheitsverhalten [26]. Vergleichbare Analysen anderer Studien liegen hierzu nicht vor.

## Limitationen

Die durchgeführte Studie hat auch Limitationen. So beruhen die Daten auf Selbstangaben der befragten Personen, sodass das Antwortverhalten durch Unwissenheit oder soziale Erwünschtheit beeinflusst werden konnte. Bei der Interpretation sollte berücksichtigt werden, dass die vorliegende Studie im Vergleich zu umfangreicheren Erhebungsmethoden in den Betrieben selbst nur geschätzte Häufigkeiten und damit nur grobe Indikatoren liefert. Dementsprechend bieten die hier vorgestellten Daten zur Inanspruchnahme aus Sicht der Bevölkerung keine Informationen über die Qualität der Angebote oder die Häufigkeit der Nutzung durch die Befragten. So ist beispielsweise bei den Angeboten zu Kantinen mit gesunden Ernährungsangeboten nicht bekannt, wie häufig die Befragten tatsächlich die gesünderen Speisenangebote gewählt haben. Die vorgenommene Einteilung der Betriebsgröße ermöglicht zwar eine differenzierte Betrachtung von kleineren Betrieben, Betriebe mit mehr als 50 beschäftigten Personen wurden hingegen nicht weiter differenziert und die Fallzahlen für Kleinstbetriebe sind gering. In ersten multivariaten Regressionsanalysen fand sich auch kein signifikanter Effekt der Betriebsgröße. Des Weiteren ist die Studie eine Querschnittsstudie, sodass die hier dargestellten Assoziationen keine kausalen Rückschlüsse erlauben.

## Schlussfolgerung

Über BGF-Maßnahmen kann ein großer Teil der Bevölkerung erreicht werden. Für die Inanspruchnahme spielen verschiedene Faktoren eine Rolle. So zeigen die Daten von GEDA 2014/2015-EHIS aus Sicht der Beschäftigten, dass deutschlandweit neben den soziodemografischen und betriebsbedingten Faktoren auch das Gesundheitsbewusstsein und die subjektive Gesundheit bei der Inanspruchnahme von Bedeutung sind. So nehmen Beschäftigte mit ausgeprägtem Gesundheitsbewusstsein alle drei Angebote häufiger in Anspruch als Beschäftigte mit weniger ausgeprägtem Gesundheitsbewusstsein. Männer mit schlechtem Gesundheitszustand nutzen die verhaltenspräventiven Angebote häufiger als Männer mit gutem Gesundheitszustand. Diese Faktoren sollten daher bei der Konzipierung von Maßnahmen berücksichtigt werden.

Insgesamt nehmen mehr Frauen als Männer die verhaltenspräventiven Angebote wahr. Dabei nehmen Frauen in Vollzeit die Angebote häufiger in Anspruch als teilzeitbeschäftigte Frauen. Diese Unterschiede im Umfang der Erwerbstätigkeit verdeutlichen die Notwendigkeit, die Bedürfnisse von Personen mit Vereinbarkeitsverantwortung stärker zu berücksichtigen ebenso wie Geschlecht und Alter sowie weitere Diversitätskategorien, wie z. B. Migrationshintergrund. Zudem sind die möglichen Barrieren für die Nutzung des Angebots einer Kantine zu prüfen und ggf. abzubauen, z. B. könnten über geringere Preise Beschäftigte mit niedrigerem Einkommen besser erreicht werden. Da die höchste Inanspruchnahme von BGF-Maßnahmen bei kleinen Unternehmen vorliegt, aber dort bisher im Vergleich zu mittleren und größeren Unternehmen weniger Maßnahmen angeboten werden, sollten diese anhand einer gezielten Informationspolitik über die Möglichkeit einer Unterstützung und Beratung durch die Krankenkassen und der Steuerfreiheit von BGF-Maßnahmen unterrichtet und ihre Ressourcen gestärkt werden. Zudem sollten die Angebote in Branchen ausgeweitet werden, in denen eine hohe Arbeitsbelastung vorliegt, wie unter anderem das Gesundheitswesen und der Bereich „Erziehung und Unterricht“. Weiterer Forschungsbedarf besteht hinsichtlich zielgruppenspezifischer Inanspruchnahme von Angeboten und Interventionen sowie einer genaueren Analyse nach Branchen. Bevölkerungsbasierte Surveys wie die GEDA-Studie ermöglichen Aussagen zur Inanspruchnahme und zu deren Faktoren aus Sicht der Beschäftigten deutschlandweit und können als Basis dienen, um die Bedarfe der verschiedenen Zielgruppen besser zu erheben und diese Forschungslücke zu schließen.

## References

[1] The Lancet Public Health (2018). Public health and the workplace: a new era dawns. Lancet Public Health.

[2] Kroll LE, Müters S, Dragano N (2011). Arbeitsbelastungen und Gesundheit.

[3] Robert Koch-Institut (2014). Gesundheitsschädigende Arbeitsbedingungen. Faktenblatt zu GEDA 2012: Ergebnisse der Studie „Gesundheit in Deutschland aktuell 2012“.

[4] Statistisches Bundesamt (2018). Mikrozensus. Bevölkerung und Erwerbstätigkeit. Stand und Entwicklung der Erwerbstätigkeit in Deutschland 2017.

[5] European Network for Workplace Health Promotion (ENWHP) (2007) Luxemburger Deklaration zur betrieblichen Gesundheitsförderung in der Europäischen Union; 2. akt. Fassung [Luxembourg Declaration on Workplace Health Promotion in the European Union; übersetzt von der Nationalen Kontaktstelle Deutschland des ENWHP]. http://www.dnbgf.de. Zugegriffen: 18. Juni 2013

[6] Faller G, Faller G (2017). Was ist eigentlich BGF?. Lehrbuch Betriebliche Gesundheitsförderung.

[7] Präventionsgesetz (2015) Gesetz zur Stärkung der Gesundheitsförderung und der Prävention Bundesgesetzblatt Jahrgang 2015 Teil I Nr 31 vom 24. Juli 2015. Bundesanzeiger Verlag, Köln

[8] Initiative Gesundheit und Arbeit (iga) (2019). Wirksamkeit und Nutzen arbeitsweltbezogener Gesundheitsförderung und Prävention. Zusammenstellung der wissenschaftlichen Evidenz für den Zeitraum 2012 bis 2018.

[9] Faller G (2018). Umsetzung Betrieblicher Gesundheitsförderung/Betrieblichen Gesundheitsmanagements in Deutschland: Stand und Entwicklungsbedarfe der einschlägigen Forschung. Gesundheitswesen.

[10] Hollederer A, Wießner F (2015). Prevalence and development of workplace health promotion in Germany: results of the IAB Establishment Panel 2012. Int Arch Occup Environ Health.

[11] Zok K, Badura B, Schröder H, Vetter C (2009). Stellenwert und Nutzen betrieblicher Gesundheitsförderung aus Sicht der Arbeitnehmer. Fehlzeiten-Report 2008 Betriebliches Gesundheitsmanagement: Kosten und Nutzen.

[12] Medizinischer Dienst des Spitzenverbandes Bund der Krankenkassen, GKV-Spitzenverband (2019). Präventionsbericht 2019. Leistungen der gesetzlichen Krankenversicherung: Primärprävention und betriebliche Gesundheitsförderung. Leistungen der sozialen Pflegeversicherung: Prävention in stationären Pflegeeinrichtungen Berichtsjahr 2018.

[13] Janßen C, Sauter S, Kowalski C (2012) The influence of social determinants on the use of prevention and health promotion services: results of a systematic literature review. GMS Psycho-Social-Med 9:Doc07 10.3205/psm000085 PMC3488803 23133501

[14] Saß A, Lange C, Finger J (2017). „Gesundheit in Deutschland aktuell“ – Neue Daten für Deutschland und Europa. Hintergrund und Studienmethodik von GEDA 2014/2015-EHIS. J Health Monit.

[15] Lange C, Finger J, Allen J (2017). Implementation of the European health interview survey (EHIS) into the German health update (GEDA). Arch Public Health.

[16] Bödeker W, Hüsing T (2008). IGA-Barometer 2. Welle. Einschätzungen der Erwerbsbevölkerung zum Stellenwert der Arbeit, zur Verbreitung und Akzeptanz von betrieblicher Prävention und zur krankheitsbedingten Beeinträchtigung der Arbeit – 2007.

[17] Schreiter I (2014). Zusammenschau von Erwerbstätigenbefragungen aus Deutschland.

[18] Robert Koch-Institut (2017). Fragebogen zur Studie „Gesundheit in Deutschland aktuell“: GEDA 2014/2015-EHIS. J Health Monit.

[19] Lampert T, Kroll LE (2014). Soziale Unterschiede in der Mortalität und Lebenserwartung.

[20] Robert Koch-Institut, Robert Koch-Institut (2015). Kapitel 3.2 Arbeit und Gesundheit. Gesundheit in Deutschland. Gesundheitsberichterstattung des Bundes.

[21] Lampert T, Kroll LE, Müters S, Stolzenberg H (2013). Messung des sozioökonomischen Status in der Studie „Gesundheit in Deutschland aktuell“ (GEDA). Bundesgesundheitsblatt.

[22] Ganzeboom HBG, De Graaf PM, Treiman DJ (1992). A standard international socio-economic index of occupational status. Soc Sci Res.

[23] Beck D, Lenhardt U, Schmitt B, Sommer S (2014). Wovon hängt die Verbreitung unterschiedlicher Niveaus betrieblicher Gesundheitsförderung ab? Vertiefende Analysen der repräsentativen Arbeitgeberbefragung 2011 für die GDA-Dachevaluation. Gesundheitswesen.

[24] Lißner L, Brück C, Stautz A, Riedmann A, Strauß A (2014). Abschlussbericht zur Dachevaluation der Gemeinsamen Deutschen Arbeitsschutzstrategie.

[25] Forschungsdatenzentren der Statistischen Ämter des Bundes und der Länder (2016) Gemeinschaftsstatistik über Einkommen und Lebensbedingungen 2013, On-Site-Zugang, Fragebogen. https://www.forschungsdatenzentrum.de/de/10-21242-63411-2013-00-00-1-1-0. Zugegriffen: 10. April 2020

[26] Jordan S, von der Lippe E (2012). Angebote der Prävention – Wer nimmt teil?.

[27] Gould SJ (1990). Health consciousness and health behavior: the application of a new health consciousness scale. Am J Prev Med.

[28] Lampert T, Schmidtke C, Borgmann LS, Poethko-Müller C, Kuntz B (2018). Subjektive Gesundheit bei Erwachsenen in Deutschland. J Health Monit.

[29] Beck D, Lenhardt U (2016). Betriebliche Gesundheitsförderung in Deutschland: Verbreitung und Inanspruchnahme. Ergebnisse der BIBB/BAuA-Erwerbstätigenbefragung 2006 und 2012. (Aktualisierung der Daten anhand der BIBB/BAuA-Erwerbstätigenbefragung 2018). Gesundheitswesen.

[30] Hurrelmann K, Kolip P, Hurrelmann K, Kolip P (2002). Geschlecht, Gesundheit und Krankheit: Männer und Frauen im Vergleich: Eine Einführung. Geschlecht, Gesundheit und Krankheit: Männer und Frauen im Vergleich.

[31] Sieverding M, Scharzer R (2005). Geschlecht und Gesundheit. Gesundheitspsychologie – Enzyklopädie der Psychologie.

[32] Grunow D, Grunow-Lutter V, Hurrelmann K, Kolip P (2002). Geschlechtsspezifische Formen von Selbstvorsorge und Selbsthilfe. Geschlecht, Gesundheit und Krankheit: Männer und Frauen im Vergleich.

[33] Setzwein M (2006). Frauenessen – Männeressen? Doing gender und Essverhalten.

[34] Mensink G, Truthmann J, Rabenberg M (2013). Obst- und Gemüsekonsum in Deutschland: Ergebnisse der Studie zur Gesundheit Erwachsener in Deutschland (DEGS1). Bundesgesundheitsblatt.

[35] Max Rubner-Institut (2008) Nationale Verzehrsstudie II – Ergebnisbericht, Teil 1. www.bmel.de/SharedDocs/Downloads/Ernaehrung/NVS_Ergebnisbericht.html. Zugegriffen: 28. Febr. 2020

[36] Max Rubner-Institut (2008) Nationale Verzehrsstudie II – Ergebnisbericht, Teil 2. www.bmel.de/SharedDocs/Downloads/Ernaehrung/NVS_ErgebnisberichtTeil2.html. Zugegriffen: 28. Febr. 2020

[37] Friedrichs M, Jungmann F, Liebermann S (2011). iga.report 21. iga-Barometer 3. Welle 2010. Einschätzungen der Erwerbsbevölkerung zum Stellenwert der Arbeit, zum Gesundheitsverhalten, zum subjektiven Gesundheitszustand und zu der Zusammenarbeit in altersgemischten Teams.

[38] Bundesagentur für Arbeit (2018). Blickpunkt Arbeitsmarkt – Die Arbeitsmarktsituation von Frauen und Männern 2017.

[39] Pöge K, Bereswill M (2019). Die doppelte Vergesellschaftung – eine Spezifik der Lebenssituation von Frauen? Gesellschaftstheoretische und wissenssoziologische Geschlechterperspektiven auf die partnerschaftliche Arbeitsteilung. Geschlecht als sensibilisierendes Konzept.

[40] Ducki A, Bamberg E, Ducki A, Metz A (2011). Gendersensible betriebliche Gesundheitsförderung. Gesundheitsförderung und Gesundheitsmanagement in der Arbeitswelt.

[41] Pieck N (2013). Gender Mainstreaming in der betrieblichen Gesundheitsförderung – Zur Bedeutung eines beteiligungsorientierten Vorgehensmodells.

[42] Bundesministerium für Arbeit und Soziales (2018). Sicherheit und Gesundheit bei der Arbeit 2017. Unfallverhütungsbericht Arbeit.

[43] Kryspin-Exner I, Pintzinger N (2010). Theorien der Krankheitsprävention und des Gesundheitsverhaltens.

[44] Seibt A, Bundeszentrale für gesundheitliche Aufklärung (BZgA) (2011). Sozial-kognitives Prozessmodel des Gesundheitsverhaltens. Leitbegriffe der Prävention und Gesundheitsförderung. Neuausgabe 2011.

[45] Faltermaier T (2011). Gesundheitsverhalten, Krankheitsverhalten, Gesundheitshandeln.

